# Non-adherence in randomised controlled trials: empirical comparison of treatment policy and efficacy estimands using individual participant data

**DOI:** 10.1186/s12874-025-02760-6

**Published:** 2026-01-24

**Authors:** Mohammod B. A. Mostazir, Joshua E. J. Buckman, Nicola Wiles, Glyn Lewis, Steve Pilling, Rob Saunders, David Kessler, Chris Salisbury, Gareth Ambler, Zachary D. Cohen, Steven D. Hollon, Simon Gilbody, Tony Kendrick, Edward Robert Watkins, William Edward Henley, Rod S. Taylor

**Affiliations:** 1 Faculty of Health and Life Sciences, School of Psychology, Mood Disorder Centre, University of Exeter, Exeter, EX4 4QG UK; 2 Centre for Outcomes Research and Effectiveness (CORE), Research Department of Clinical, Educational & Health Psychology, University College London, London, WC1E 7HB UK; 3 Bristol Medical School, Centre for Academic Mental Health, Population Health Sciences, University of Bristol, Canynge Hall, 39 Whatley Road, Bristol, BS8 2PS UK; 4 Centre for Academic Primary Care, Bristol Medical School, Population Health Sciences, University of Bristol, Canynge Hall, 39 Whatley Road, Bristol, BS8 2PS UK; 5 Department of Statistical Science, University College London, Gower Street, London, WC1E 6BT UK; 6 Department of Psychology, University of Arizona, Tucson, United States of America; 7 Department of Psychology, Vanderbilt University, Nashville, TN 37023 United States; 8 Hull York Medical School and Department of Health Sciences, University of York, York, United Kingdom; 9 Primary Care Research Centre, Population Sciences & Medical Education, University of Southampton, Aldermoor Health Centre, Primary Care, Southampton, SO16 5ST United Kingdom; 10 Department of Health and Community Sciences, Medical School, University of Exeter, University of Exeter, Exeter, EX1 2LU UK; 11 MRC/CSO Social and Public Health Sciences Unit & Robertson Centre for Biostatistics, Institute of Health and Well Being, University of Glasgow, Glasgow, G2 3AX UK

**Keywords:** Individual patient data (IPD) meta-analysis, Non-adherence, Intention-to-treat, Per-protocol (PP), Complier average causal effect (CACE), Estimands, Randomised controlled trials (RCTs)

## Abstract

**Background:**

Non-adherence to interventions is common in randomized controlled trials (RCTs), complicating the interpretation of treatment effects. The intention-to-treat (ITT) principle estimates the treatment effect of assignment to intervention but does not reflect efficacy among those who adhere. Per-protocol (PP) analyses attempt to address this but introduce selection bias by violating randomisation. The complier average causal effect (CACE) provides an efficacy estimand among compliers while preserving randomisation. This study aimed to provide an empirical comparison of ITT, PP, and CACE approaches using individual participant data (IPD) from trials of depression interventions in primary care.

**Methods:**

We analysed IPD from the Depression in General Practice (Dep-GP) collaboration, comprising seven eligible RCTs with 3,467 participants. Trials reported continuous (depression symptom scores) or binary (treatment response) outcomes. Adherence was defined within the intervention group. We conducted a two-stage IPD meta-analysis to estimate treatment effects under ITT, PP, and CACE. Results were expressed as differences in standardised mean difference (ΔSMD) for continuous outcomes and as ratios of odds ratios (ROR) for binary outcomes. One-stage mixed-effects models were performed as secondary analyses.

**Results:**

For binary outcomes, both PP and CACE analyses produced larger effects than ITT (ROR for PP vs ITT: 1.09; 95% CI, 1.05–1.14; *P* < .001; CACE vs ITT: 1.19; 95% CI, 1.00–1.42; *P* < .05). For continuous outcomes, CACE yielded a larger effect than ITT (ΔSMD = 0.10; 95% CI, 0.01–0.20; *P* < .05), while PP did not differ from ITT (ΔSMD = 0.03; 95% CI, –0.01 to 0.08; *P* = .167). Sensitivity analysis, excluding the TREAD trial, yielded larger effect by the PP method (ΔSMD = 0.05; 95% CI, 0.01–0.09; *P* < .05).

**Discussion:**

Our findings demonstrate that CACE provides a causal efficacy estimand that diverges from the treatment policy effect estimated by ITT, while PP yields similar but potentially biased results. This highlights the importance of distinguishing between estimands in the presence of non-adherence and illustrates empirically how they differ in practice.

**Conclusions:**

Current RCT reporting recommendations should be updated to require routine reporting of CACE alongside ITT, together with adherence information, to provide a more complete and transparent account of treatment effects.

**Supplementary Information:**

The online version contains supplementary material available at 10.1186/s12874-025-02760-6.

## Introduction

Evidence for the effectiveness of interventions should ideally come from randomised controlled trials (RCTs). However, non-adherence to intervention protocol is a common issue in RCTs. Such non-adherence may arise due to factors including but not limited to, missed treatment schedules, unwillingness to receive the intervention, or adverse effects associated with the intervention. The ICH E9(R1) addendum [1] on estimands and sensitivity analyses has emphasised the importance of clearly defining the *treatment effect of interest*—the estimand—before turning to the choice of estimator or analysis method. In this framework, the conventional intention-to-treat (ITT) analysis corresponds to a treatment policy estimand, addressing the question: *What is the effect of assigning treatment, regardless of whether participants adhere?* The Consolidated Standards of Reporting Trials (CONSORT) statement recommends analysing outcomes based on the ITT principle, i.e., comparing participants in the intervention and control groups according to their original randomised allocation, regardless of their adherence to the intervention or whether they actually received it [2].

By preserving the original randomisation, ITT maintains baseline prognostic balance, minimises selection bias and provides a valid estimate of treatment policy estimand. However, when adherence is low, ITT may not reflect the efficacy of the intervention, since it averages outcomes over both ‘compliers’ and ‘non-compliers’ [3]. Alternative estimands are therefore sometimes of interest. To account for the effects of non-adherence, an on-treatment estimand, per-protocol (PP) analysis is often conducted to compare outcomes between the control group and a subset of participants in the intervention group who adhered to a pre-defined intervention protocol [4]. However, the PP approach is prone to selection bias and confounding [5, 6], as it no longer maintains the balance achieved through randomisation of trial participants. As a result, PP method may overestimate the treatment effect and increase the risk of type-I error by rejecting the null hypothesis incorrectly.

A more rigorous alternative is the complier average causal effect (CACE), which targets a principal stratum estimand: the effect of treatment among those who would comply with their assignment, whether allocated to intervention or control. CACE preserves randomisation through instrumental variable methods and provides a causal estimate of efficacy in the presence of non-adherence [7]. While CACE has been proposed as a complementary approach that closely estimates intervention efficacy in the presence of non-adherence [8, 9], its estimation procedure can be complex or even infeasible in studies with multiple intervention arms and may rely on some assumptions that are not empirically verifiable [10, 11].

Despite the ICH framework clarifying these distinctions, many RCTs continue to report PP analyses—sometimes alongside ITT, and sometimes without acknowledging the corresponding estimand. For example, a review of RCTs published between 1991 and 2015 showed that 56% of trials reported their outcome results using PP analysis [12]. Similar findings were reported by a review of 100 RCTs published during 2008 that found 47% reporting a PP analysis [13]. Moreover, a meta-analysis [14] of trial-level data from 156 RCTs published in high impact general medical journals (The Lancet, New England Journal of Medicine, British Medical Journal, Journal of American Medical Association, and The Annals of Internal Medicine) between 2017 and 2019 showed that reported PP estimates were, on average, 2% greater than ITT estimates (ratio of odds ratios (ROR):1.02, 95% confidence interval (CI): 1.00 to 1.04, *P* = 0.03). The divergence between PP and ITT estimates tended to increase with higher levels of non-adherence to the intervention protocol. However, such trial-level meta-analysis and comparison of statistical methods relied on published results, which may reflect heterogeneous analytic choices.

To provide a more consistent empirical comparison of estimands, individual participant data (IPD) meta-analysis enables estimation of ITT, PP, and CACE effects across trials using uniform analytic approaches [15]. Our study therefore undertook an IPD meta-analysis of depression trials in primary care to compare intervention effects under these three estimands. We hypothesised that, in the presence of non-adherence, both CACE and PP would yield larger estimated effects than ITT. Our findings aim to illustrate, in practice, how alternative estimands diverge, and to demonstrate the importance of aligning estimand choice with the clinical or policy question at hand.

## Methods

### Data source

We used a previously compiled IPD dataset comprising 11 RCTs, curated for the Depression in General Practice (Dep-GP) project [16, 17]. The Dep-GP collaboration comprises randomised controlled trials of adults (≥16 years) with depression who sought treatment in primary care. The collaboration investigated the association between socioeconomic factors and depressive symptoms (measured using BDI-II and PHQ-9), irrespective of treatment type, using an individual participant data (IPD) meta-analysis of 4,864 participants (mean [SD] age 42.5 [14.0] years; 3,279 women [67.4%]). The study reported that depressive symptom scores were 28% (95% CI, 20%–36%) higher among unemployed participants than among those employed, and 18% (95% CI, 6%–30%) lower among homeowners compared with participants living with family or friends, in hostels, or homeless, independent of the treatments they received. We provided the relevant citations for interested readers who wish to explore the Dep-GP project in more detail. We used this dataset because: (i) it includes a group of RCTs conducted in primary health care settings and testing a variety of widely used treatments for depression, all using common depression outcome measures—e.g., Patient Health Questionnaire-9 (PHQ-9) or Beck Depression Inventory–II (BDI-II) and, (ii) it potentially included trials that collected and reported a quantitative measure of intervention adherence. Chief investigators of all included trials were contacted and invited to share their original trial datasets for the present study.

### Definition of ITT, PP, and CACE population

The ITT population was defined as: all eligible participants who were randomised to treatment arms and outcomes were observed. The PP population was defined as: all participants in the intervention group who adhered to the treatment protocol, where adherence was set to a minimum level of intervention defined by the original trial investigators or based on a common standard that the original investigators used. Level of adherence was also considered for including participants in the PP population where an active control group was present and a standard for minimum adherence to active treatment was defined by the trial investigators. CACE analysis included the same population as ITT analysis but, we used the term ‘compliers’ to indicate those in the intervention group who were offered treatment and adhered to treatment protocol, and ‘non-compliers’ to indicate those in the intervention group who were offered treatment but failed to adhere to treatment protocol.

### Data preparation

We reanalysed outcome data from each included trial and cross-checked it against the original publications. All datasets were reviewed for completeness and consistency in key variables including demographic characteristics, stratification or minimisation variables, intervention adherence measures, and missing observations, to ensure that all values were representative of the original studies. For trials that tested the hypothesis of a worse outcome (higher score in depression), intervention groups were reversed so that the direction of between-group effects was consistent across all trials. Any discrepancies, missing data, or adherence-related information were verified and resolved with the original trial authors and the chief investigator of the Dep-GP trials, who is the data custodian.

### Outcomes

Of the seven included trials, the primary outcome for GENPOD, MIR and TREAD was the continuous BDI-II measure, COBALT and IPCRESS was the binary versions of the BDI-II (COBALT: ≥50% reduction from baseline; IPCRESS: BDI-II<10 at follow-up), PANDA was the continuous PHQ-9, and HEALTHLINES was the binary version of the PHQ-9 measure defined as a PHQ-9 score <10 and a reduction of ≥5 points from baseline. In all cases, authors who used continuous measures as their primary outcome also reported a binary version for secondary analyses, so a dichotomised definition of the outcome was available for all trials. For our one-stage IPD meta-analysis, the continuous measures from PHQ-9 and BDI-II were standardized to z-score within each trial. For all studies except HEALTHLINES, we used the primary endpoint as the trial outcome. Although the HEALTHLINES trial specified a 4-month primary endpoint, it conducted CACE analyses at 12 months. To ensure comparability between CACE and ITT estimates, we treated the 12-month outcome as the primary endpoint for the HEALTHLINES trial.

### Statistical analyses

#### Descriptive analysis

Demographic and trial related categorical variables (e.g. gender, history of depression, ethnicity, treatment groups, publication year, intervention type, non-adherence, outcome type) are presented as frequencies and percentages. Continuous variables (e.g. follow-up duration, baseline/follow-up outcomes are presented as mean and standard deviation (SD). Estimated intervention effects using ITT, PP, CACE methods are presented for each trial as unstandardized or standardized mean difference (Hedge’s-g) and odds ratios (OR).

#### Primary analysis

Random effect two-stage IPD meta-analysis method using restricted maximum likelihood (REML) estimation were applied for both continuous and binary outcomes (PHQ-9 or BDI-II) to compare CACE, PP, and ITT estimates. The choice of a two-stage approach over a one-stage model was pragmatic, given that trials used different outcome measures (PHQ-9 and BDI-II). The two-stage method allowed us to estimate trial-level intervention effects on the original outcome scale before pooling and comparing the estimates across analytic methods. Additionally, the two-stage approach enabled replication of the analytical models used in the original trials, including adjustment for trial-specific stratification and minimisation variables. In contrast, the one-stage method required standardising outcome measures to z-scores and did not allow for inclusion of stratification variables that were not common to all studies. For these reasons, one-stage models were included as part of our secondary analyses. Since ITT, PP, and CACE estimates are sourced from the same participants on the same outcome, standard errors for their differences were adjusted assuming strong within-study correlation. The complier average causal effect (CACE) was estimated using instrumental-variable (IV) approach implemented via structural equation modelling (SEM) framework. Randomisation served as the instrument, influencing the outcome only through participants’ compliance behaviour. Detailed statistical methods are presented in the online supplementary document (eAppendix-1). Generic example code illustrating the implementation of the instrumental-variable CACE estimation used in this study is provided in (eAppendix-3) of the Supplement, as the exact specification varies by trial depending on available adherence and covariate data.

#### Secondary analyses

Secondary analyses included comparisons of ITT, PP, and CACE estimates using a one-stage method with random-effects models for both continuous and binary outcome measures. Methods for comparing between the estimates for the secondary analyses applying one-stage method are presented in the online supplementary document (eAppendix-1).

### Meta-regression and sub-group analyses

Meta-regression was used to explore the impact of trial level covariates i.e., non-adherence rate, trial duration, intervention type on divergence of treatment effects for PP and CACE compared to ITT. Further sensitivity analyses were carried out by adjusting standard errors assuming different within trial correlations. Sub-group analyses were carried out to explore heterogeneity.

For all models, pooled ITT, PP, and CACE estimates and their differences are presented. For continuous outcomes, the estimates are presented with Hedge’s-g effect size. For pooled ITT/PP/CACE models with binary outcome, effect sizes are presented with odds ratio (OR) and ratio of odds ratios (ROR) are presented for their differences. Statistical significances are presented with p-values and heterogeneity statistics *I*^*2*^ and *tau*^*2*^ are presented. Statistical significance was assessed at 5% level. All data curation and analyses were carried out in statistical software Stata (version-18) [18].

All data were pseudo-anonymised and securely hosted in the UCL’s Data Safe Heaven (DSH) server in Stata format. To ensure data security, downloading from the DSH server to local computer was restricted, and all analyses were conducted within the DSH environment.

## Results

### Eligible studies

**Fig. 1:**
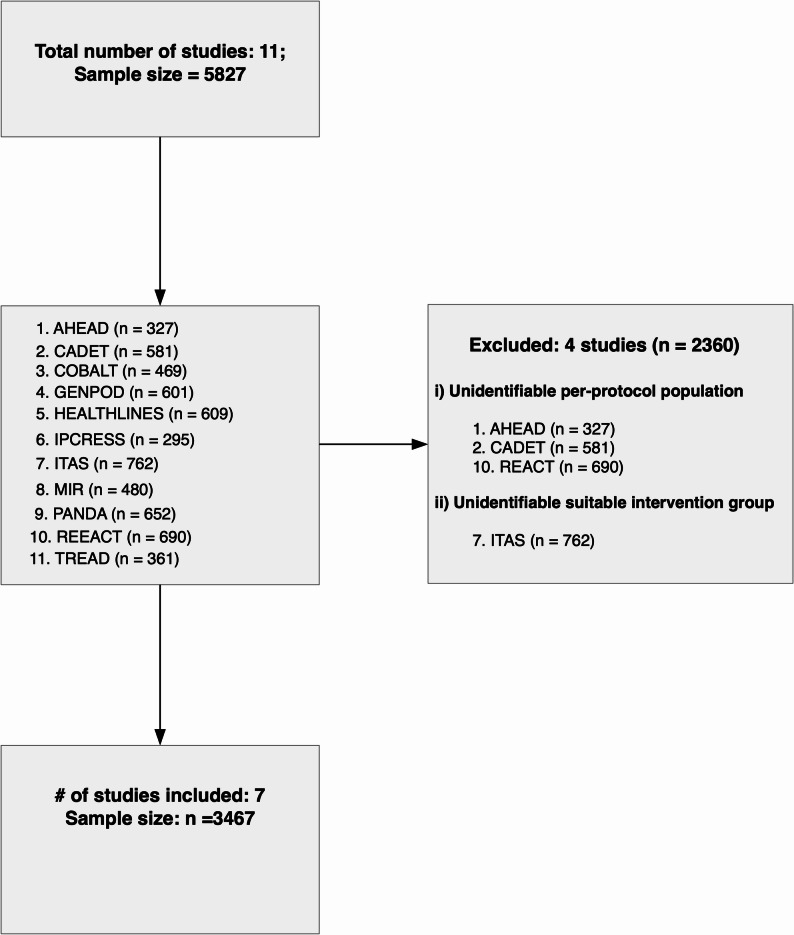
Study selection flowchart

All trial teams responded positively to our request to use their data in the present study and seven [19–25] out of a total of eleven RCTs were deemed eligible (Figure 1). Three RCTs (AHEAD, REEACT, and CADET) [26–28] were excluded because they did not quantify intervention adherence. One trial (ITAS) [29] was ineligible due to the absence of a suitable intervention group. Figure-1 presents the selection process.

### Descriptive participant and trial statistics

All studies were RCTs investigating effect of antidepressants (*n* = 3), cognitive behavioural therapy (CBT) (*n* = 2), telehealth care (*n* = 1), or physical activity promotion (*n* = 1) intervention on the outcome of depression (see Table 1). A total of 3467 trial participants in primary care were randomised to one of two arms across seven studies. Follow-up information on outcomes was available from 85% (*n* =2957) of those randomised. The majority (67%) of trial participants were female (66–72%), mean age ranging from 39 to 51 years, with 95% having a white ethnic background. Trial outcome follow-up duration used here ranged from 6 to 26 weeks. BDI-II was used as the primary outcome for 5/7 studies. Adherence information was available for all patients in the intervention except HEALTHLINES (*n* = 6), MIR (*n* = 32), and PANDA (*n* = 66) for whom adherence information was missing. Mean adherence in the intervention group across studies was 73% (range 57% to 94%).

**Table 1:** Study characteristics, mean/SDs of outcomes and treatment effects for all 7 studies

Study characteristics	COBALT	GENPOD^5^	HEALTHLINES	IPCRESS	MIR	PANDA	TREAD	Overall^3^
Authors/year	Wiles et. al., 2013	Lewis et. al., 2011	Salisbury et. al., 2016	Kessler et. al., 2009	Kessler et. al., 2018	Lewis et. al. 2019	Chalder et. al. 2012	
Outcome^1^	BDI-II	BDI-II	PHQ-9	BDI-II	BDI-II	PHQ-9	BDI-II	–
Intervention type	CBT^2^	Antidepressant	Tele health care	CBT	Antidepressant	Antidepressant	Physical activity	–
*Sample characteristics*								
Control: n(%)	235 (50)	303 (50)	302 (50)	147 (50)	239 (50)	329 (50)	179 (50)	1734 (50)
Intervention: n(%)	234 (50)	298 (50)	307 (50)	148 (50)	241 (50)	323 (50)	182 (50)	1733 (50)
Total sample: n(%)	469 (100)	601 (100)	609 (100)	295 (100)	480 (100)	652 (100)	361 (100)	3467 (100)
Compliers n(%)^2^	144 (62)	239 (80)	218 (72)	90 (61)	151 (72)	241 (94)	103 (57)	1186 (73)
Duration in weeks^3^	26	6	17	17	12	6	17	14
Female: n (%)	339 (72)	408 (68)	417 (69)	200 (68)	332 (69)	384 (59)	237 (66)	2317 (67)
Age (years): mean (SD)	50 (12)	39 (12)	50 (13)	35 (12)	51 (13)	40 (15)	40 (13)	44 (14)
Ethnicity (White): n (%)	459 (98)	575 (96)	592 (98)	281 (95)	468 (98)	579 (89)	336 (93)	3290 (95)
History of depression: n (%)	415 (89)	434 (72)	527 (92)	227 (77)	396 (83)	522 (80)	254 (70)	2775 (81)
*Baseline outcome score: n*	469	601	609	295	480	650	361	3465
Control: mean (SD)	31.83 (11)	33.41 (10)	16.68 (5)	33.46 (9)	30.64 (10)	12.20 (6)	32.07 (10)	32.26 (10);^a^ 14.34 (6)^b^
Intervention: mean (SD)	31.76 (10)	33.94 (9)	17.06 (5)	32.87 (8)	31.48 (10)	11.76 (6)	32.05 (9)	32.48 (10);^a^ 14.35 (6)^b^
Overall: mean (SD)	31.79(11)	33.67 (10)	16.87 (5)	33.16 (9)	31.06 (10)	11.98 (6)	32.06 (9)	32.37 (10);^a^ 14.35 (6)^b^
*Follow-up outcome score: n*	419	546	516	206	431	551	288	2957
Control: mean (SD)	24.51 (13)	19.58 (11)	11.89 (6)	22.09 (13)	19.69 (12)	8.75 (6)	16.87 (13)	20.55 (13);^a^ 10.25 (6)^b^
Intervention: mean (SD)	18.94 (14)	18.87 (11)	11.56 (6)	14.51 (11)	17.97 (12)	7.98 (6)	16.12 (11)	17.75 (12);^a^ 9.73 (6)^b^
Overall: mean (SD)	21.77 (14)	19.22 (11)	11.73 (6)	17.97 (13)	18.54 (12)	8.38 (6)	16.50 (12)	19.15 (12);^a^ 10.00 (6)^b^
*Binary outcome: no/yes*^*4*^								
Control: n (%)	167/46 (78/22)	211/61 (78/22)	175/86 (67/33)	73/21 (78/22)	139/78 (64/36)	121/164 (42/58)	94/52 (64/36)	980/508 (66/34)
Intervention: n (%)	111/95 (54/46)	214/60 (78/22)	160/95 (63/37)	70/42 (62/38)	119/95 (56/44)	97/169 (36/64)	102/40 (72/28)	873/596 (59/41)
Overall: n (%)	278/141 (66/34)	425/121 (78/22)	335/181 (65/35)	143/63 (69/31)	258/173 (60/40)	218/333 (40/60)	196/92 (68/32)	1853/1104 (63/37)
*Treatment effects (continuous)*^*6*^								
ITT: *B* (95% CI)	−5.68 (−7.95 to −3.41)	−1.19 (−2.90 to 0.52)	−0.51 (−1.52 to 0.49)	−7.04 (−10.05 to −4.03)	−1.86 (−3.95 to 0.23)	−0.51 (−1.33 to 0.31)	−0.54 (−3.06 to 1.98)	–
Effect size: Hedge's-g	−0.42	−0.11	−0.08	−0.57	−0.15	−0.09	−0.04	–
PP: *B* (95% CI)	−7.47 (−9.91 to −5.03)	−1.08 (−3.00 to 0.85)	−0.87 (−1.95 to 0.21)	−7.47 (−10.87 to −4.08)	−2.25 (−4.67 to 0.16)	−0.63 (−1.47 to 0.21)	0.07 (−2.90 to 3.04)	–
Effect size: Hedge's-g	−0.56	−0.10	−0.14	−0.60	−0.18	−0.11	0.01	–
CACE: *B* (95% CI)	−8.53 (−12.13 to −4.93)	−1.08 (−3.22 to 1.06)	−0.61 (−1.98 to 0.77)	−11.41 (−16.43 to −6.39)	−2.55 (−5.41 to 0.32)	−0.66 (−1.53 to 0.21)	−1.16 (−5.58 to 3.25)	–
Effect size: Hedge's-g	−0.64	−0.10	−0.10	−0.92	−0.20	−0.11	−0.09	–
*Treatment effects (binary)*^*6*^								
ITT: OR (95% CI)	3.30 (2.12 to 5.13)	1.06 (0.70 to 1.61)	1.23 (0.85 to 1.78)	2.43 (1.24 to 4.78)	1.43 (0.96 to 2.12)	1.24 (0.84 to 1.82)	0.67 (0.40 to 1.12)	–
PP: OR (95% CI)	3.76 (2.32 to 6.08)	1.11 (0.70 to 1.77)	1.40 (0.95 to 2.08)	2.85 (1.37 to 5.96)	1.55 (0.99 to 2.42)	1.28 (0.85 to 1.92)	0.68 (0.37 to 1.24)	–
CACE: OR (95% CI)	6.64 (4.01 to 11.02)	1.17 (0.77 to 1.79)	1.29 (0.89 to 1.9)	2.75 (1.42 to 5.31)	1.69 (0.98 to 2.94)	1.28 (0.84 to 1.93)	0.71 (0.39 to 1.27)	–

^1^*BDI-II* Beck depression inventory II, *PHQ-9* Patient Health Questionnaire-9, *CBT* Cognitive behavioural therapy

^2^Compliers indicate only those in the intervention group and adhered to treatment protocol; Compliance information was missing for HEALTHLINES (n = 6), MIR (n = 32) PANDA (n = 66)

^3^For the Overall column: Duration in weeks = mean weeks; Outcome = ^a^Mean BDI-II; ^b^mean PHQ-9

^4^Binary outcome represents number of patients did not improve/improved (%) at follow-up in each group according to the trial’s definition of dichotomised outcome

^5^For GENPOD, Citalopram (SSRI) group was used as the intervention group to estimate treatment effects

^6^Models are adjusted for baseline score; B = unstandardized mean difference; OR = odds ratio of patient improvement; Stratification/minimisation variables: COBALT: councillor, antidepressant, duration depression; GENPOD: severity of symptom; HEALTHLINES: general practitioner service; IPCRESS: antidepressant, sex, councillor, depression; MIR: sex, receiving therapy; PANDA: depression severity/duration; TREAD: antidepressant; depression severity, physical activity

Table 1 presents estimated treatment effects and effect sizes (Hedge’s-g/OR) for each trial using ITT, PP, and CACE methods on both binary and continuous measures. Only one trial (MIR) reported estimates from PP analysis in their published report, and 5 trials (except GENPOD and PANDA) reported CACE estimates besides ITT analysis. On the continuous outcome, our PP estimates were of a greater magnitude than the ITT estimates for all trials except GENPOD and TREAD. In the CACE method the estimated treatment effects were larger than estimates from the ITT method, for all trials. For binary outcomes, both CACE and PP produced larger treatment effect than ITT for all trials.

### Primary analysis

**Fig. 2:**
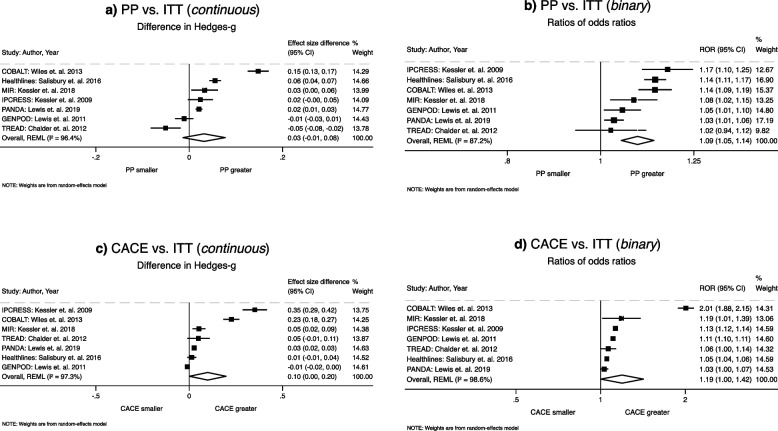
Primary analyses (two-stage method): (**a**) PP vs. ITT on continuous outcome; (**b**) PP vs. ITT on binary outcome; (**c**) CACE vs. ITT on continuous outcome (**d**) CACE vs. ITT on binary outcome

Primary analyses results are presented in Table 2 and the results of pooled ITT, PP, and CACE models on binary and continuous outcomes. In the two-stage model with continuous outcome, the pooled intervention effect estimated using the ITT method was SMD = −0.19 (95% CI: −0.33 to −0.06; *P* < 0.01; see Table 2), indicating an overall intervention effect in reducing depression at follow-up. The treatment effect estimated by the PP method was SMD = −0.23 (95% CI: −0.40 to −0.07; *P* < 0.01). There was no evidence that PP estimates differed from the ITT estimates (ΔSMD = 0.03; 95% CI: −0.01 to 0.08; *P* = 0.167; Table 2, Fig. 2a). One trial (TREAD) produced a smaller PP estimate compared to ITT (Fig. 2a). Sensitivity analysis excluding the TREAD trial, the difference between PP and ITT estimates became statistically significant (ΔSMD = 0.05; 95% CI: 0.01 to 0.09; *P* = 0.043).

**Table 2:** Comparisons of pooled treatment effects estimated by ITT, PP, and CACE analysis methods

Outcome	Meta-analysis models^e^	Pooled ITT				Pooled PP				Pooled CACE			
		*ES* (95% CI)	*P*	*I*^*2*^	*τ*^*2*^	*ES* (95% CI)	*P*	*I*^*2*^	*τ*^*2*^	*ES* (95% CI)	*P*	*I*^*2*^	*τ*^*2*^
*Pooled analyses*													
Continuous (SMD)^a^	Two-stage	−0.19 (−0.33 to −0.06)	0.004	69.18	0.02	−0.23 (−0.40 to −0.07)	0.006	75.95	0.04	−0.29 (−0.52 to −0.06)	0.012	86.62	0.08
Continuous (SMD)^a^	One-stage	−0.18 (−0.28 to −0.07)	0.001	–	–	−0.21 (−0.34 to −0.07)	0.003	–	–	−0.23 (−0.39 to −0.07)	0.004	–	–
Binary (OR)^b^	Two-stage	1.41 (0.97 to 2.07)	0.074	80.70	0.21	1.55 (1.03 to 2.32)	0.036	79.51	0.23	1.70 (1.00 to 2.88)	0.050	88.04	0.45
Binary (OR)^b^	One-stage	1.40 (1.02 to 1.92)	0.035	*–*	–	1.54 (1.11 to 2.14)	0.010	–		1.56 (1.25 to 1.94)	0.000	–	–
Difference between methods		PP vs. ITT				CACE vs. ITT				CACE vs. PP			
		Difference (95% CI)	*P*	*I*^*2*^	*τ*^*2*^	Difference (95% CI)	*P*	*I*^*2*^	*τ*^*2*^	Difference (95% CI)	*P*	*I*^*2*^	*τ*^*2*^
*Primary analyses*													
Continuous (ΔSMD)^c^	Two-stage	0.03 (−0.01 to 0.08)	0.167	98.01	0.00	0.10 (0.01 to 0.20)	0.047	99.39	0.02	0.07 (−0.02 to 0.16)	0.135	99.90	0.01
Binary (ROR)^d^	Two-stage	1.09 (1.05 to 1.14)	0.000	84.61	0.00	1.19 (1.00 to 1.42)	0.048	99.90	0.05	1.09 (0.93 to 1.29)	0.280	99.74	0.05
*Secondary analyses*													
Continuous (ΔSMD)^c^	One-stage	0.03 (0.001 to 0.06)	0.023	–	–	0.06 (0.01 to 0.11)	0.032	–	–	0.02 (0.00 to 0.05)	0.046	–	–
Binary (ROR)^d^	One-stage	1.10 (1.08 to 1.12)	0.000	–	–	1.11 (1.01 to 1.22)	0.025	–	–	1.01 (0.91 to 1.13)	0.843	–	–

*ITT* Intention to trea, *PP* Per-protocol, *CACE* Compliers average causal estimate,* I*^*2*^ and* τ*^*2*^ presents heterogeneity statistic

^a^SMD = Standardized mean difference (Hedge's-g) presented as effect size (ES)

^b^OR = Odds ratio (OR) for binary outcome pooled model for ITT, PP, CACE methods

^c^ΔSMD = Difference between standardized mean difference between estimation methods

^d^ROR = Ratio of odds ratio as a difference between two odds ratios from estimation methods

^e^All two-stage meta-analyses models used restricted maximum likelihood (REML) technique and all one-stage models are random effect model

The pooled treatment effect estimated using the CACE method was SMD = −0.29 (95% CI: −0.52 to −0.06; *P* = 0.012). The estimate was larger than the ITT estimate (ΔSMD = 0.10; 95% CI: 0.01 to 0.20; *P* = 0.047; Table 2, Fig. 2c). There was no evidence for a difference between the CACE and PP estimates (ΔSMD = 0.07; 95% CI: −0.02 to 0.16; *P* = 0.135; Table 2).

With the binary outcome, using the two-stage method, the pooled ITT estimate was OR = 1.41 (95% CI: 0.97 to 2.07; *P* = 0.074), and the pooled PP estimate was OR = 1.55 (95% CI: 1.03 to 2.32; *P* = 0.036; Table 2). The PP method produced a 9% larger effect size compared to ITT (ROR = 1.09; 95% CI: 1.05 to 1.14; *P* < 0.001; Table 2, Fig. 2b).

The pooled CACE estimate was OR = 1.70 (95% CI: 1.00 to 2.88; *P* = 0.051), which was larger than the ITT estimate (ROR = 1.19; 95% CI: 1.00 to 1.42; *P* = 0.048; Table 2, Fig. 2d). There was no difference between the CACE and PP estimates (ROR = 1.09; 95% CI: 0.93 to 1.29; *P* = 0.280; Table 2).

### Secondary analyses

The pooled ITT estimate for the continuous outcome using a one-stage random-effects model was SMD = −0.18 (95% CI: −0.28 to −0.07; *P* = 0.001). The corresponding PP estimate was SMD = −0.21 (95% CI: −0.34 to −0.07; *P* = 0.003), which was larger than the ITT estimate (ΔSMD = 0.03; 95% CI: 0.001 to 0.06; *P* = 0.023) (Table 2). The pooled CACE estimate from the one-stage model was SMD = −0.23 (95% CI: −0.39 to −0.07; *P* < 0.01), which was also larger than the ITT estimate (ΔSMD = 0.06; 95% CI: 0.01 to 0.11; *P* = 0.032). The CACE estimate was also greater than the PP estimate (ΔSMD = 0.02; 95% CI: 0.00 to 0.05; *p* = 0.046)

The pooled ITT effect for binary outcome with one-stage random effect model was OR: 1.40, 95% CI: 1.02 to 1.92, *P* = 0.035. The corresponding pooled PP estimate was OR: 1.54, 95% CI: 1.11 to 2.14, *P* = 0.010. The difference indicated larger estimates for PP method (ROR: 1.10, 95% CI: 1.08 to 1.12, *P* < 0.001). The pooled OR for CACE estimate was 1.56, 95% CI: 1.25 to 1.94, *P* < 0.001. The estimate was larger than the ITT estimate (ROR: 1.11, 95% CI: 1.01 to 1.22, *P* = 0.025). There was no evidence of CACE estimate being different than PP estimate (ROR: 1.01, 95% CI: 0.91 to 1.13, *P* = 0.843).

### Between trial heterogeneity, risk of bias and meta-regression

Substantial statistical heterogeneity (*I*^*2*^) was observed across the two-stage models reflecting differing trial populations and interventions. In the pooled ITT, PP, and CACE models, the two CBT trials — COBALT and IPCRESS — showed notably larger effect sizes compared to the others (Table 1). Subgroup analyses by intervention type showed that effect sizes differed between CBT and non-CBT trials (*P* < 0.001). Other subgroup analyses — including intervention type (antidepressant vs. others), non-adherence proportion, and trial duration — did not explain the observed heterogeneity.

In the pooled difference models, the *I*^*2*^ statistics were high primarily because the differences between the standard errors of two estimates were small, resulting in a very low *τ*^*2*^ (between-study variance). As part of sensitivity analyses, we re-estimated the standard errors under various assumed correlations between the estimates, ranging from *r* = 0.50 ~ 1.00. For continuous outcome analysed with two stage models, the statistical significance of the differences between PP vs. ITT, and PP vs. CACE remained unchanged across all assumptions (all *P*>0.05). Statistical significance for the CACE vs. ITT comparison was also maintained (*P*<0.05) for most assumptions. However, the *I*^*2*^ value decreased as weaker correlation was assumed. Notably, for PP vs. ITT and PP vs. CACE models, *I*^*2*^ dropped to 0.01% when the assumed correlation was as low as *r* = 0.70, and for CACE vs. ITT models, *I*^*2*^ decreased to 37%. Similar patterns were observed for models with binary outcomes. Study-specific variance ratio tests comparing the ITT and PP samples were all non-significant, with large P-values (all *P* > 0.70), indicating no evidence of unequal variances (eAppendix-2). Egger’s test [30] showed no evidence (*P*>0.05) of small study/publication bias and meta-regression did not show any meaningful associations to the differences between the treatment effects.

## Discussion

This IPD meta-analysis compares intervention effect estimates under intention-to-treat (ITT), complier average causal effect (CACE), and per-protocol (PP) analyses using data from 2,957 participants across seven RCTs. Our results show that both CACE and PP produce larger treatment effects compared to ITT across primary two-stage and secondary one-stage analyses for both continuous and binary outcomes. However, all three estimands (ITT, PP, and CACE) produced consistent conclusions regarding statistical significance across all seven trials. That is, when the ITT effect was statistically significant, so were the PP and CACE estimates and vice versa (Table 1).

These findings are consistent with our earlier trial level meta-analysis, which showed that the PP and CACE methods yield larger treatment effects than ITT, and that CACE and PP estimates are similar in magnitude [31]. When non-adherence obscures the true causal effect of treatment, PP is often used alongside ITT. Although reporting of PP is common and despite being prone to bias, this is often not recognised by investigators. In our previous study, we found that investigators in 37% of trials used PP estimates to confirm the robustness of the trial findings, with statements such as: *“Analysis of the primary outcome in the per-protocol population confirmed this result” or “We undertook a per protocol analysis for the primary outcome to check the robustness of conclusions”* [31]. Given the susceptibility of PP analyses to selection bias, this widespread reliance can be misleading and may adversely influence clinical judgement. The ICH E9(R1) addendum explicitly notes that PP sets may not align with any meaningful estimand and are subject to severe bias, since excluding non-compliers after randomisation breaks the balance that underpins causal inference [1]. For this reason, PP cannot be recommended as a framework for addressing causal questions.

By contrast, CACE provides a principled alternative approach when an efficacy estimand is of interest in the presence of non-adherence, whereas ITT appropriately targets the treatment policy (effectiveness) estimand. CACE estimates the effect among the principal stratum of compliers, preserving randomisation through instrumental variable methods. This makes CACE a more appropriate alternative to PP in the context of non-adherence and retaining a clear causal interpretation. Thus, whilst PP and CACE point estimates appear similar in our dataset, they differ fundamentally in their inferential validity and interpretation [7, 32]. However, when adherence is low, CACE estimates can become unstable, potentially yielding inflated treatment effects with large standard errors [33]. Furthermore, the binary concept of ‘acceptable adherence’ can be somewhat arbitrary when the amount of intervention received is a continuous variable. Therefore, the definition of adherence should be pre-specified, and the threshold chosen should be justified, ideally during the trial design phase.

A key contribution of our study is to provide an empirical demonstration of how ITT and CACE estimands diverge in practice. While theoretical arguments are well rehearsed, practical illustrations remain scarce. By applying a consistent analytic framework across multiple RCTs, we show the degree of divergence between treatment policy and efficacy estimands and highlight the importance of specifying the estimand at the design stage.

In summary, our findings support routine reporting of both ITT and CACE in RCTs where non-adherence is present. ITT remains the gold standard for estimating the treatment policy estimand, while CACE provides a causal efficacy estimand among compliers. Together, these complementary perspectives can provide a more transparent and informative basis for interpreting trial results.

### Strengths and limitations

To our knowledge, this is the first study to use IPD meta-analysis to directly compare the intervention effects of different statistical estimation methods in RCTs in the presence of non-adherence. However, this study has some potential limitations. First, the included data set focused on mental health intervention and outcomes which may limit the generalisability of our findings to other RCT disease areas. Second, our statistical models showed considerable statistical heterogeneity reflecting the variation in trial populations, interventions, and comparators. However, given the aim of this study was not to provide a definitive estimate of treatment effects but to compare different statistical analysis approaches, we believe this is not a major limitation. However, to account for statistical heterogeneity we used random effects models. The adjustment of standard errors based on a strong assumed correlation may have also contributed to observed heterogeneity. Nevertheless, variance ratio tests showed that the variances of ITT and PP samples were similar supporting our correlation assumption. Finally, we did not include analyses based on the ‘as-treated method’ [4], as this approach compromises randomisation and causal inference.

### Implications for future trials

Our findings have important implications of specifying and reporting estimands in RCTs, particularly in the context of intervention non-adherence. Whilst the ITT approach remains the gold standard for estimating treatment policy estimand, it reflects the impact of treatment assignment rather than underlying therapeutic efficacy. In contrast, the PP method, though often used in practice to assess efficacy, is subject to selection bias and can yield misleading conclusions. The CACE estimand offers a more reliable alternative by estimating the causal effect of treatment among compliers, while retaining the integrity of the randomised design. Therefore, trials should report both ITT and CACE analysis, together with clear adherence information, so that results can inform both policy decisions and efficacy assessments. In addition, documenting reasons for treatment discontinuation or modification should be reported as recommended by Standard Protocol Items: Recommendations for Interventional Trials (SPIRIT) 2025 (item 15b) [34] and Consolidated Standards of Reporting Trials (CONSORT) 2025 (item 24a) [2]. Furthermore, incorporating CACE estimates alongside ITT results in evidence synthesis and guidelines could offer a more comprehensive understanding of treatment effects, particularly in real-world settings where optimal perfect intervention adherence is infrequently achieved.

## Conclusions

This IPD meta-analysis study showed that CACE estimates of treatment efficacy were consistently larger than ITT estimates of the treatment policy effect, while PP produced similar but biased results. These findings reinforced the need to distinguish between policy and efficacy estimands in the presence of non-adherence. Given its causal basis and preservation of randomisation, current RCT reporting recommendations should be updated to routinely include reporting of CACE alongside ITT, together with adherence information, to provide a more complete and transparent account of treatment effects.

## Supplementary Information

Supplementary Material 1.

## Data Availability

This study was based on individual participant data from multiple randomised controlled trials. Data access permissions were granted for the purposes of this specific study, with approvals obtained individually from the data custodians of each trial. The datasets are therefore not publicly available but may be made available upon reasonable request to the corresponding author and with appropriate permissions from the relevant data authorities.

## Abbreviations

CACE: Complier average causal effect
CI: Confidence interval
ΔSMD: Difference in standardised mean difference
IPD: Individual participant data
ITT: Intention-to-treat
PP: Per-protocol
RCT: Randomised controlled trial
ROR: Ratio of odds ratios
