# Supplementary material for: Non-adherence in randomised controlled trials: empirical comparison of treatment policy and efficacy estimands using individual participant data
Source: BMC Med Res Methodol. 2026 Jan 24;26:40. doi: 10.1186/s12874-025-02760-6 (PMC12914939; doi:10.1186/s12874-025-02760-6)
Supplement: Supplementary file 1 — Supplementary Material 1. [file 12874_2025_2760_MOESM1_ESM.docx]

**Contents:**

- **Appendix-1: Methods of statistical analysis**
- **Appendix-2: Variance ratio tests between ITT and PP samples**
- **Appendix-3: Generic Stata codes for CACE implementation**

**Appendix-1: Methods of statistical analysis**

**Primary analysis**

**i. Comparison of PP vs. ITT using two-stage IPD meta-analysis with continuous outcome:** To compare the intervention effect between PP vs. ITT for each trial*_i_*_,_ a linear regression model was developed to estimate the treatment effect using the ITT method that was originally employed by individual trials with adjustment for baseline score of the primary outcome and trial specific stratification/minimisation variables. The adjusted treatment effect i.e., (Δ*_itti_* = adjusted group difference between treatment and control groups) was converted to a standardised mean difference (SMD = Hedge’s g = Δ*_ittdi_*) using the samples used in the regression models and their within group observed standard deviations.^32^ The same procedure was applied for deriving treatment effect for the PP population (Δ*_ppi_*) followed by converting the Δ*_ppi_* to SMD (Δ*_ppdi_*). Further, the difference (𝜽*_i_*) between the two SMD treatment effects (Δ*_ppdi_* and Δ*_ittdi_*) for each trial was calculated allowing the difference (𝜽*_i_*) to have a positive (+) sign if PP method produced a larger effect and a (-) sign if PP method produced smaller effect compared to ITT. Since 𝜽*_i_* is the difference between two estimators (ITT and PP) sourced from same trial and essentially from the same outcome and same participants, the two treatment effects were inextricably linked. The standard error of 𝜽*_i_* for each trial, therefore, was adjusted using the observed pooled correlation (*r* ) of the two effect sizes across studies using the formula^32^:

$$\omega_{i}= \sigma_{itti}+\sigma_{ppi} -2r \sqrt{\sigma_{itti}} \sqrt{\sigma_{ppi}}$$

where, $\omega_{i}$ is the estimate of variance for each trial, $\sigma_{itti}$ is the variance from the ITT sample, $\sigma_{ppi}$is the variance from the PP sample and *r* is the correlation coefficient for the two estimates. The square root of the variance $(\sqrt{\omega_{i}})$ provided the standard error (σ*_i_*) for each trial. The correlations between ITT and PP estimates across studies was nearly perfect *r* = ~0.99, which is similar to what we observed in our previous study^13^ with aggregated trial level data with 156 studies (*r* = ~0.97). We therefore adjusted the standard errors as if the ITT and PP are perfectly correlated. As an additional check to ensure that the variances of the ITT and PP groups were derived from the same population, we conducted variance ratio tests^33^ for each trial. After estimating the trial level estimates (𝜽*_i_*, σ*_i_*), restricted maximum likelihood (REML) based random effect meta-analysis was conducted using the ‘meta’ suite of commands in the statistical software Stata^17^. The overall pooled summary effect represented the magnitude of extent to which PP estimates are smaller/greater compared to the ITT estimates.

**ii. Comparison of ITT vs. CACE using two-stage IPD meta-analysis with continuous outcome:** To compare CACE and ITT estimates, CACE was estimated for each trial using the instrumental variable (IV) method via the generalised structural equation modelling (GSEM) framework with an identity link function. Randomisation was used as the instrument, and adherence information from the intervention group was treated as the endogenous variable to estimate CACE. Error variance of adherence and the outcome was allowed to covary. To convert CACE estimates into standardised effect sizes (Hedges’ g) for the two-stage meta-analysis, the observed standard deviation of the intervention group sample used in the GSEM model was applied. Since the CACE framework assumes that the proportion of compliers is the same in the control group as in the intervention group, the sample size for compliers in the control group was estimated as 𝜋 x n, where 𝜋 is the proportion of compliers in the intervention group, and n is the control group sample size used in the GSEM model. The corresponding observed standard deviation for this subset was then used for standardisation. After converting the CACE estimates into standardised effect sizes, comparison to ITT estimates was made following similar procedures explained earlier.

**iii. Two-stage IPD meta-analysis with binary outcome (PP vs. ITT vs. CACE)**: To compare the estimates on binary outcome, trial specific ITT and PP estimates (OR = odds ratios) were obtained applying logistic regression models adjusted for the baseline score and stratification/minimisation variables. The comparison of the two estimates followed similar procedures as planned with continuous outcome i.e., estimating trial level difference (𝜽*_i_*) between the two estimates (𝜽*_i_ = ln_PPi_*  – *ln_ITTi_* ) and adjustment of standard error for within trial correlation, except no standardisation of the treatment effect was required. The ratio of odds ratio (ROR) was calculated as the exponent of the pooled difference (𝜽*_i_*) between the two logged effect estimates (*ln_PP_*  – *ln_ITT_* ) across studies, providing an estimate of how much PP estimates are greater/smaller compared to the ITT estimates. Similar process was followed for making comparison with CACE estimates except odds ratios for CACE estimates were obtained by instrumental variable approach via generalised structural equation modelling (GSEM) with a logit link for the dichotomised outcome. Latent variables were used to covary the error variance between adherence and the binary outcome.

**Secondary analysis**

**i. One-stage IPD meta-analysis using binary/continuous outcome (PP vs. ITT vs. CACE):** Unlike the two-stage IPD method, which uses trial level information, the one-stage method used all individual patient-level data available from the selected trials to estimate the overall intervention effect and make comparisons between PP, ITT and CACE estimates. For the one-stage meta-analysis with binary outcome, maximum likelihood (ML) based mixed effect logistic regression models were fitted separately using ITT and PP sample to estimate the pooled treatment effect *ln*(OR) for both ITT ${(\delta}_{itt}$) and PP sample ${(\delta}_{pp}$). In addition to within trial standardised baseline score of the outcome variables, models included trial specific random intercepts to account for trial level heterogeneity and fixed effect for centres. Robust standard errors were estimated using the heteroskedasticity-consistent (HC) estimator. Since the stratification/minimisation variables varied from one trial to another, one-stage analyses models did not include those variables except ‘sites of recruitment’ which was common to all studies. The two pooled estimates ${(\delta}_{itt}, \delta_{pp}$) were tested for equality applying a Z-test with test statistic:^34,35^

$$Z=\frac{\propto}{\sqrt{SE{pp}^{2}+SE{itt}^{2}-2r*SEpp*SEitt}}$$

where, ∝ is the difference between the two estimates from ITT and PP model and *SEpp* and *SEitt* are the standard errors from the two estimation methods. The standard errors in the dominator were also adjusted for within trial correlated outcomes. The exponent of ∝ represented the ratio of the odds ratios (ROR) of greater/smaller PP estimates compared to ITT. 95% confidence interval (CIs) of the difference were calculated using the combined adjusted standard error of the difference quantity ∝ ±1.96*SE. Same procedure was followed to compare the CACE estimates against ITT/PP estimates except CACE estimates were obtained with instrumental variable approach applying generalised estimating equation (GSEM) with a logit link function for the binary outcome and accounted for trial-level heterogeneity. Similar process of comparison was followed for the one-stage model with continuous measure except the outcome measures (PHQ-9/BDI-II) were standardised into z-score within trial so that the intervention effect represents overall between-group standardised mean difference (SMD).

**Appendix-2: Variance ratio tests between ITT and PP samples**

| Study | ITT (n) | Variance ITT | *df*-1 | PP (n) | Variance PP | *df*-2 | F-statistic | *P-values* |
| --- | --- | --- | --- | --- | --- | --- | --- | --- |
| COBALT | 419 | 193.335 | 418 | 355 | 187.114 | 354 | 1.033 | 0.751 |
| GENPOD | 546 | 120.652 | 545 | 441 | 121.422 | 440 | 0.994 | 0.942 |
| HEALTHLINES | 516 | 39.609 | 515 | 458 | 40.162 | 457 | 0.986 | 0.877 |
| IPCRESS | 206 | 164.209 | 205 | 172 | 170.238 | 171 | 0.965 | 0.802 |
| MIR | 431 | 153.282 | 430 | 327 | 150.123 | 326 | 1.021 | 0.845 |
| PANDA | 551 | 33.116 | 550 | 508 | 32.689 | 507 | 1.013 | 0.883 |
| TREAD | 288 | 143.889 | 287 | 235 | 151.417 | 234 | 0.950 | 0.679 |

ITT = Intention-to-treat; PP = Per-protocol; n: = Sample, *df* = degrees of freedom

**Appendix-3: Generic Stata codes for study specific CACE implementation via instrumental variable (IV) method using Generalised structural equation model (GSEM)**

1. **^*^For continuous outcome:**
   - comp = compliance (0 = ‘no’, 1 = ‘yes’)
   - trt = randomisation (0 = ‘control’, 1 = ‘intervention’)
   - y1 = follow-up outcome
   - y0 = baseline outcome score
   - adjustment_variables = study-specific stratification or covariate adjustment set

#delimit ;

gsem (comp <- trt, fam(gaus))

(y1 <- y0 comp adjustment_variables, fam(gaus))

cov(e.comp*e.y1)

;

#delimit cr

1. **^*^For binary outcome:**
   - comp = compliance indicator (0 = no, 1 = yes)
   - trt = randomisation (0 = control, 1 = intervention)
   - binout = binary follow-up outcome
   - y0 = baseline outcome score
   - adjustment_variables = study-specific stratification or covariate adjustment set
   - L1, L2 = latent variables allowing the error terms of the continuous endogenous compliance variable and the binary outcome to covary

#delimit ;

gsem (comp <- trt L@1, fam(gaus))

(binout <- y0 comp adjustment_variables L2@1, fam(bin))

var(e.comp) cov(L2 L1) diff intpoints(12) intmethod(mcaghermite)

iterat(1000) technique(nr 10 bfgs 10)

;

#delimit cr

** Model convergence issues may arise depending on sample size, degree of non-adherence, and covariate structure. The above code provides a generic template only.*
